# Localize.pytom: a modern webserver for cryo-electron tomography

**DOI:** 10.1093/nar/gkv400

**Published:** 2015-04-30

**Authors:** Thomas Hrabe

**Affiliations:** Bioinformatics and Systems Biology, Sanford-Burnham Medical Research Institute, La Jolla, CA 92037, USA

## Abstract

Localize.pytom, available through http://localize.pytom.org is a webserver for the localize module in the PyTom package. It is a free website and open to all users and there is no login requirement. The server accepts tomograms as they are imaged and reconstructed by Cryo-Electron Tomography (CET) and returns densities and coordinates of candidate-macromolecules in the tomogram. Localization of macromolecules in cryo-electron tomograms is one of the key procedures to unravel structural features of imaged macromolecules. Positions of localized molecules are further used for structural analysis by single particle procedures such as fine alignment, averaging and classification. Accurate localization can be furthermore used to generate molecular atlases of whole cells. Localization uses a cross-correlation-based score and requires a reference volume as input. A reference can either be a previously detected macromolecular structure or extrapolated on the server from a specific PDB chain. Users have the option to use either coarse or fine angular sampling strategies based on uniformly distributed rotations and to accurately compensate for the CET common ‘Missing Wedge’ artefact during sampling. After completion, all candidate macromolecules cut out from the tomogram are available for download. Their coordinates are stored and available in XML format, which can be easily integrated into successive analysis steps in other software. A pre-computed average of the first one hundred macromolecules is also available for immediate download, and the user has the option to further analyse the average, based on the detected score distribution in a novel web-density viewer.

## OVERVIEW

### Introduction

The 3D structure of intact macromolecules provides valuable information for functional analysis when molecules are imaged *in situ*. This information is essential because it allows not only to understand pathways within healthy organisms but also to analyse macromolecular processes that lead to diseases.

Cryo-Electron Tomography (CET) is the method that allows 3D imaging of macromolecules *in situ* at the highest resolutions currently possible (currently ∼50^−1^Å^−1^). Coherent averaging of many macromolecule densities imaged inside the cytosol or attached to organelles typically improves the spatial resolution of a molecule under scrutiny to 10^−1^–30^−1^Å^−1^ and allows detailed analysis of its structure (1,2). Recent instrumental advances in Cryo-Electron Microscopy (CEM), such as direct detector cameras and phase plates, significantly increased the image quality of micrographs and conclusively final resolution of averages in the last years (3,4). Ion milling of cryo samples allows creating thin lamellas of regions in the cellular environment that would remain inaccessible otherwise (5). Mapping averages back to their original coordinates in tomograms allows one to create 3D molecular atlases of cells or organelles with potentially various types of macromolecules (6,7).

Localize.pytom.org is a new webserver developed for the specific purpose of accurately detecting macromolecules in tomograms.

### Method

Robust localization of macromolecules in electron tomograms by template matching was introduced more than a decade ago (8) and re-implemented (9) based on the PyTom platform (10). A small template (or reference) is rotated in all possible 3D orientations and each orientation is searched in the larger tomogram, using the locally normalized cross-correlation. High correlation values are indicative of macromolecules with a high similarity to the reference. One key improvement implemented for localization was the use of equidistant sampling angles determined by quaternions. Localization by template matching is a considerably CPU and memory expensive task and requires parallel processing to sample all possible orientations of the template inside the tomogram. The server currently running localization uses 10 CPUs for computation.

### Comparison

While cryo-electron microscopy and tomography completed an evolutionary leap in terms of data quality in the last decade, method development hardly kept pace with modern concepts in software development. Thus far, all methodological developments in CET (and CEM) have been focussed on the implementation of full pipelines EMAN2 (11), SPARX (12) , PyTom (10) or application-specific, stand-alone programs Dynamo (13), PEET (14) or JSubTomo (15). Many come with OS native user interfaces EMAN2 (11), Xmipp (16), TOM (17) or are integrated into meta-platforms for processing such as APPION (18). However, no CEM/CET method has ever been made available as an online service. To date, only the PDBe web-page offers two services to analyse data: (i) to determine the Fourier Shell Correlation and consequently the approximate resolution in a dataset (http://www.ebi.ac.uk/pdbe/emdb/validation/fsc/) and (ii) to validate tilt pairs in single particle processing (http://www.ebi.ac.uk/pdbe/emdb/validation/tiltpair/).

Webservers are a standard tool in bioinformatics that allow easy access to a large collection of methods for both users and developers. Once users have decided about a method by testing the webserver, they can proceed to download and install this particular software on their machines. This approach is not available in CEM/CET. Non-software experts are more or less confined to pre-installed software in their labs or heavily depend on their system administrator to try new methods. Even worse, developing within proprietary environments can force users to purchase those in order to use particular methods. Installation of platforms may take up a long time and platforms can prove as not suitable for the problem at hand after the first tries, what makes finding a matching platform extremely tedious. Hence, methods available through webservers can be benchmarked online prior to installation, provide an easier review process during publication and generally a wider accessibility to users.

## SERVER WORKFLOW

### Sample data

**Figure 1.**
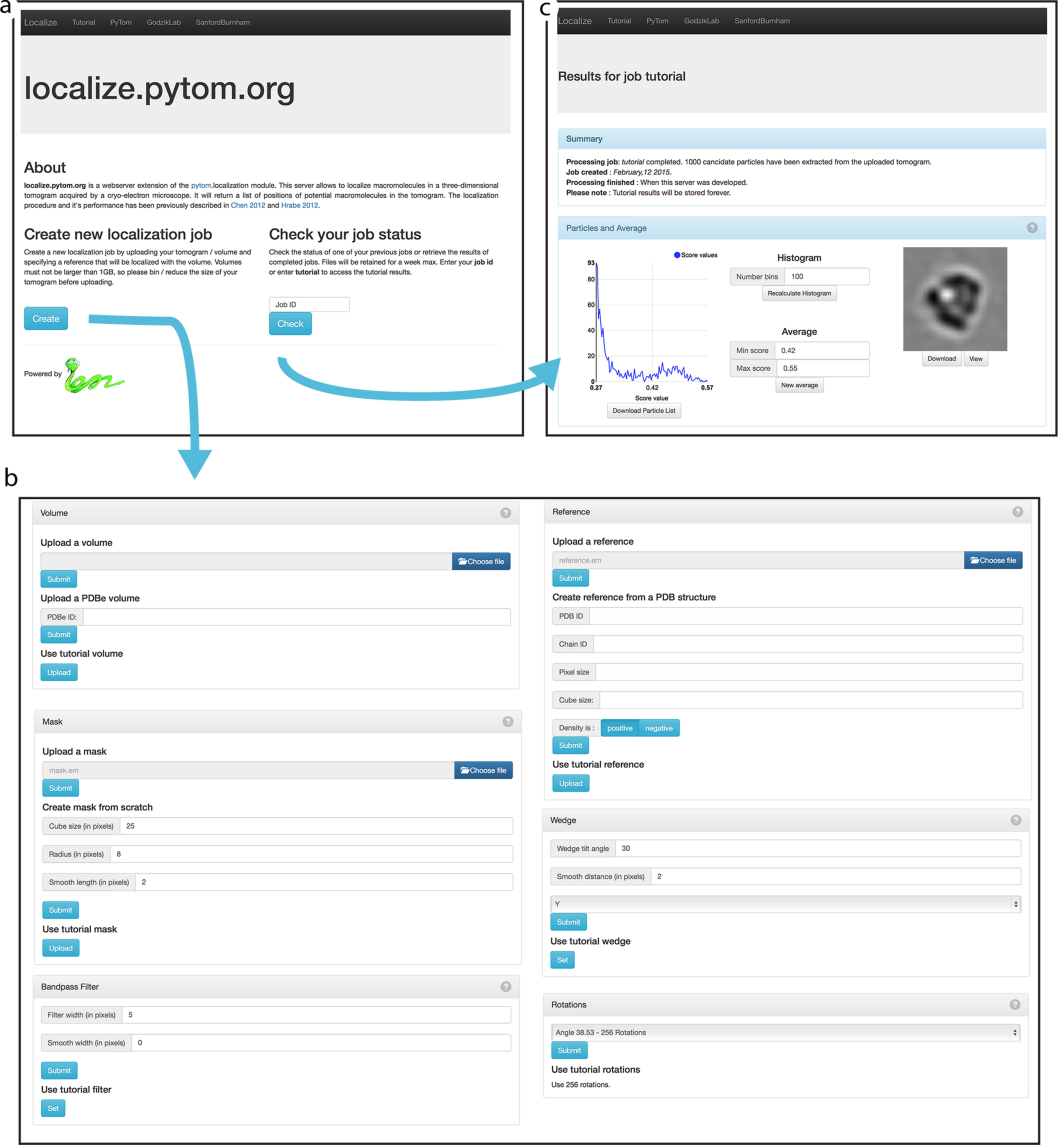
The workflow of localize.pytom.org: (**a**) the front page provides direct access to either setting up a new job (‘Create’ button) or checking back on a submitted job (‘Check’ button). The ‘Create’ button leads to the ‘new job’ page (**b**) where volumes are uploaded and runtime parameters are specified. The respective parameters for setting up jobs are described in more detail in the main text. Every form has an online help, accessible through the question mark symbol on the upper right corner. A unique job ID is assigned to each job and the user must remember this ID to access his or her results later. If the user wants to check on the status of his job, he has to input the respective job ID on the front page. Depending on the job status the user will either be forwarded to the result page or an overlay text will inform the user on whether the job is still queued or currently running. (**c**) The result page contains a rough summary of the job such as the deployment date and when it will be removed from the server. Jobs will be kept for 7 days after completion. Below is an interactive view on the localized macromolecules and links to download all result files.

In the following paragraphs a tutorial dataset previously published in PyTom (10) will be used. One tomogram of this dataset imaging complete 80S Ribosomes in a lysate (128 MB) was selected to demonstrate the workflow on the webserver (Figure 1).

### Starting a job

By clicking on the ‘new job’ button on the front page, the user will be forwarded to multiple input fields to specify various parameters for processing. Each input interface features an online documentation accessible through the ‘question mark’ symbol in the upper right corner.

A unique job ID will be provided for the user for a successful return to the server. In order to conserve space on the server, results will be kept for 7 days and deleted thereafter.

#### Data types

The server generally supports the MRC, CCP4 and EM file types used in the field. Most importantly, the server is compatible with tomograms from the EMDB/PDBe webpage to enable direct access to other resources in the near future. All volumes are converted into EM during processing and results will be available in EM format.

#### Volume upload

Uploading tomograms to the server is the primary step for creating a job on the server. Tomogram size, bandwidth and distance from the server influence the upload speed of the file. Uploads of the tutorial tomogram resulted in an ‘inter-continental’ upload time of a few minutes with a rather standard bandwidth (DSL).

Overall, the file size per tomogram is limited to 1 GB and large tomograms will be automatically binned after upload. Binning strategies are shown in Table 1. The user must upload all data in consistent voxel size. Binning will be applied to volume, reference and mask consistently. However, the user should upload either truncated or pre-binned volumes to avoid potential binning artefacts during the procedure, especially for the reference and mask.

**Table 1.** Listed here are tomogram sizes for which binning will be applied to tomogram, reference and mask

Volume size	Automatic binning factor
≤256 MB	0×
256–512 MB	1×
512 MB–1 GB	2×

Using binning ensures that the machine will never exceed the available memory during processing. However, users should truncate their tomogram to the interesting region and reduce memory consumption rather than forcing the server to bin all files and proceed at a lower resolution than necessary.

#### Reference upload or from PDB

The default reference density of an 80S Ribosome molecule is available for the tutorial. Voxel size of 18.8^−1^Å^−1^ matches the tutorial tomogram, the cube size of 25 pixels results to a total file size of 60 KB.

Jobs can be specified with any density and are accepted as a reference whenever they are cubic and do not exceed the maximum file size of 64 MB. Please note that the tutorial reference can theoretically be used with any other tomogram. However, the pixel size of the tomogram must match 18.8 ^−1^Å^−1^ to provide reasonable results if users decide to re-use the tutorial reference.

If no reference density is available, the interface offers the option to extrapolate a density volume from an existing protein structure chain stored in PDB format. For this, the user must specify the PDB ID, the chain name, the size of the resulting cube and the voxel size. The density centre of the chain will be placed into the centre of the volume. Again, it is critical that the voxel size of the tomogram and the reference are consistent to guarantee valid results.

#### Mask upload or initialization

A mask must be used to eliminate cropping artefacts when the reference is rotated. The mask cube must have the same size as the reference volume. It must be circular and should extend as far as possible to cover the whole reference density. The user can upload a mask or generate it on the fly through the webserver. Here, the user must specify the cube size, the diameter for the full mask and a Gaussian smoothing at the edge of the mask. The sum of the mask radius and the smoothing edge should not exceed half the cube size but should rather be a little bit smaller. A mask matching the tutorial reference is available and will be automatically copied to the tutorial job.

#### Wedge

The wedge information of the tomogram has to be specified by the opening angle. The tutorial dataset, for instance, was tilted from approximately −60° to 60°. Hence, an opening angle of 30º must be specified for the wedge (90º − 60º). The sampled wedge region should be smoothed by the smoothing parameter. This feature will create a Gaussian drop-off around the otherwise sharp boundaries. The smooth parameter must be specified in voxels.

PyTom assumes the Y-axis to be the default tilt axis, which is consistent with the tutorial dataset. However, this may vary of from lab to lab due to different image acquisition and volume reconstruction schemes and the order voxels are stored per file type. Depending on the uploaded data, the tilt axis can hence be specified to either X, Y or Z in PyTom specification.

Dual-tilt wedge correction is available in the PyTom package in a beta state and still needs thorough testing. Hence, it is not yet available on the webserver, but will be made available once it has been proven to produce robust results.

#### Rotations

Five rotation lists are available to specify rotation sampling during localization. The shortest list contains 100 rotations. It should be used for testing purposes only while the largest list contains 1944 rotations that allow sampling at an angular distance of 19°. The default list for the tutorial example is the list containing 256 rotations at an angular distance of 38.5°.

Using these quaternion-based lists not only improves the sampling quality compared to exhaustive sampling of three Euler angles but also significantly speeds up localization by a factor of 1.5 (10).

#### Low pass filter

Suppressing high-frequent noise using a low pass filter is a standard approach in CET and available on the webserver. Here, the highest allowed frequency band can be specified in voxels. All values up to this frequency will equally contribute to localization. The filter width can be adjusted manually in a similar way as it is done in the adaptive filtering during sub-tomogram alignment in (10). The whole filter kernel can be extended with a short drop-off for smoothing purposes of the kernel (also specified in voxels). The tutorial setting here is 5 voxels for the kernel width and a 2 voxel smoothing.

### Output

#### XML files

The most re-useable result of the server is the particle list in standard PyTom XML format. It stores the coordinates and orientations of each potential macromolecule in the analysed tomogram. The origin of the coordinates is in the centre of the tomogram and rotations are stored in the ZXZ Euler rotation paradigm. The rotation parameters store the angles required to rotate the reference into to orientation of the tomogram.

#### Macromolecular densities

All found macromolecules are available for download in a ZIP archive and can be inspected visually on the page with the new online density viewer. Each molecule is a sub-volume cut from the original volume at the assigned coordinate. The size of each cube matches the reference cube and is hence 25 voxels for the tutorial results.

#### Result density volumes

To complete the availability of all interesting files generated at runtime, the volumes of maximum scores at each position in the tomogram and the index of the corresponding rotation are available for download separately.

#### Average

An average of all macromolecules is available for download from the result page. The orientations used for averaging are the ones detected during the localization process and stored in XML files. The first average displayed on the result page is created by the first 100 macromolecules by default. However, the user can interactively adjust the number of macromolecules used for averaging in a score histogram by selecting them based on lower and upper score values. This feature can significantly improve the quality of the average (19).

For example, for the tutorial the initial average contains a gold label visualized by the exceptionally dark spot in the position of the 40S sub-unit. However, when the user specifies a custom interval for averaging to the Gaussian peak of the score histogram [0.42;0.55], no gold labels are visible in the average. The resolution for the tutorial dataset of all, unclassified macromolecules was detected to 43^−1^ Å^−1^.

### Visualization

A novel method for 3D density visualization is accessible from the result page. This technology is based on the current development process of the Chimera platform to provide an interactive visualization suite for webpages (20).

**Figure 2.**
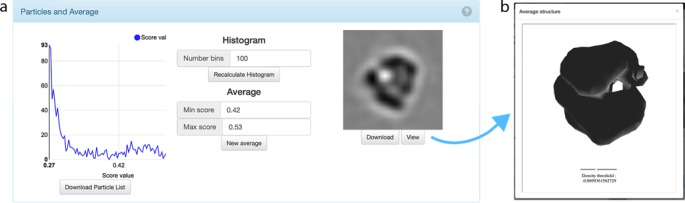
(**a**) The score histogram that indicates the distribution of score values contains 10 bins by default and was adjusted to 100 bins in this figure. The slice through the average on the right has been generated from all macromolecules detected in the score interval of 0.42–0.53. This interval can be selected interactively and the average will be updated based on the currently selected values. The full particle list and the average are available for download. (**b**) Displaying average densities is implemented using the modern WebGL technique. A Chimera process on the server creates ten density models that are displayed online in the viewer. Sliding through the density thresholds allows users to comfortably assess structure quality and localization results in a way familiar to the standard density viewer of Chimera, without having to download the average and start Chimera on their computer.

A connection from the result page to Chimera on the server allows one to display density in real time by using the modern WebGL standard for display (Figure 2). The viewer is implemented in a similar way as the density viewer in Chimera to allow users to quickly inspect the outcome without having to download the average. A slider allows the user to adjust the surface threshold to inspect a subset of all theoretically possible thresholds in Chimera; only 10 thresholds of almost infinite possibilities are available.

## DISCUSSION

This paper presents a first ever implementation of the processing of the webserver localize.pytom.org in CET. The implementation of localize.pytom.org indicates that the bandwidth threshold to transfer and process tomograms in reasonable times online has been surpassed. Web technologies have become an extremely robust interface and visualization standard in our daily life and this webserver demonstrates that an HTML-based user interface JQuery and the modern visualization technology WebGL can easily be adapted as a platform for CEM/CET data webservers. Furthermore, web technologies theoretically would also allow to implement CEM / CET cloud services in the near future, which would considerably decrease costs in computational infrastructure, especially important for smaller labs.

While a webserver or a cloud will never replace a fully established CET processing pipeline due to the benefit of physical proximity for big data processing, it does increase the accessibility of a specific method to the broad audience. The availability of more of such processing servers would allow users to rapidly evaluate multiple methods on the same data and decide which method fits their particular problem best.

A future add-on to the server would be a link to download tomograms directly from the EMDB/PDBe database (21). An interacting network of CEM/CET webservers or web services would foster the standardization of data types and parameter conventions and allow to prototype new, interesting data-processing pipelines.
